# Supplementary material for: Scalable data harmonization for single-cell image-based profiling with CytoTable
Source: Patterns (N Y). 2026 Mar 30;7(5):101514. doi: 10.1016/j.patter.2026.101514 (PMC13161684; doi:10.1016/j.patter.2026.101514)
Supplement: Document S2. Article plus supplemental information [file mmc2.pdf]

Patterns

Resource

Scalable data harmonization for single-cell image-based profiling with CytoTable

Graphical abstract

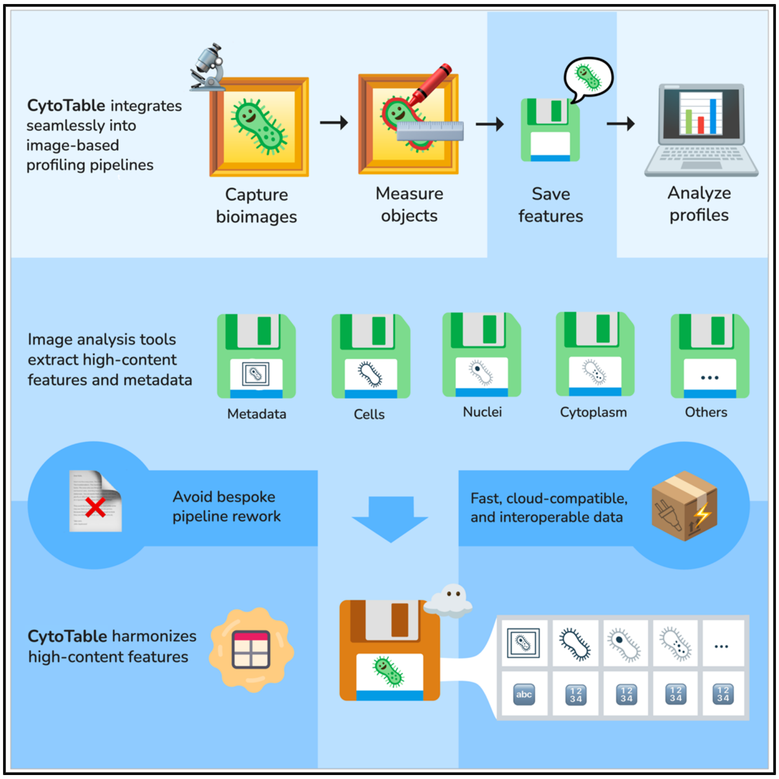

Highlights

- CytoTable standardizes single-cell morphology readouts into a common data structure
- CytoTable processes outputs from tools like CellProfiler, DeepProfiler, and IN Carta
- CytoTable outputs parquet or AnnData file formats, ready for downstream processing

Authors

Dave Bunten, Jenna Tomkinson, Erik Serrano, ..., Vince Rubinetti, Faisal Alquaddoomi, Gregory P. Way

Correspondence

gregory.way@cuanschutz.edu

In brief

High-content imaging enables large-scale phenotypic discovery, but its biological value is often limited by fragmented single-cell data organization. CytoTable addresses this challenge by providing a standardized, harmonized foundation emphasizing consistent structure, explicit data types, and fast modular integration. This approach reduces technical artifacts, improves reproducibility, and allows researchers to focus on biological questions, supporting reliable pattern discovery and collaborative analysis as microscopy datasets grow in scale and complexity.

Bunten et al., 2026, Patterns *7*, 101514
May 8, 2026 © 2026 The Author(s). Published by Elsevier Inc.
https://doi.org/10.1016/j.patter.2026.101514

CellPress

Patterns

CellPress
OPEN ACCESS

Resource

Scalable data harmonization for single-cell image-based profiling with CytoTable

Dave Bunten,[1] Jenna Tomkinson,[1] Erik Serrano,[1] Michael J. Lippincott,[1] Kenneth I. Brewer,[2] Vince Rubinetti,[1] Faisal Alquaddoomi,[1] and Gregory P. Way[1,3,*]
[1]Department of Biomedical Informatics, University of Colorado Anschutz, Aurora, CO 80045, USA
[2]Seqera Labs S.L., Barcelona, Spain
[3]Lead contact
*Correspondence: gregory.way@cuanschutz.edu
https://doi.org/10.1016/j.patter.2026.101514

SUMMARY

High-content imaging (HCI) involves the automated acquisition and quantitative analysis of cell phenotypes from microscopy images. These studies often rely on screening, which can involve thousands of chemical or genetic perturbations that produce terabytes of microscopy data. To extract meaningful biological insights, these data must be processed into quantitative features through a technique known as image-based profiling. A major analytical bottleneck is curating the high-dimensional, single-cell data derived from various image-analysis tools. These datasets suffer from inconsistent schemas, inefficient file formats, and undocumented ontological relationships. These challenges reduce reproducibility and slow progress in downstream applications. To solve these issues, we introduce CytoTable, a software package for harmonizing single-cell image-based profiling. CytoTable enables modular, portable, and cross-language data integration through a robust, reproducible, and scalable engine that harmonizes single-cell readouts from multiple image-analysis tools, preparing for feature integration with software in the Cytomining ecosystem such as Pycytominer.

INTRODUCTION

Image-based profiling is a critical part of bioinformatics processing for high-content imaging (HCI) experiments. HCI uses a pipeline of software tools: each tool reads and processes the output of the one before it, then passes its results on to the next stage (Figure 1A).[1] These data-processing pipelines start with wet lab assays and microscopy imaging devices that produce microscopy images, which are then analyzed with image-analysis tools to produce structured, high-content morphology feature measurements. Scientists then use the feature data to find patterns and test hypotheses on specific biological processes and the impact of perturbations on cell phenotypes. Recent review articles have covered these applications comprehensively.[2,3] Critical software tools and workflows for executing this complex pipeline are designed and used by bioimage analysts,[4] research software engineers,[5] and others with similar roles.

Image-based profiling data formats and structures are myriad; there is little cohesion on file type, column names, or structure. These data also tend to be "wide," entailing many thousands of columns, which makes for the high-content nature of the approach. Comma-delimited spreadsheets (e.g., CSV and TSV) or other text-based data are commonplace and ill suited to handle large data needs. For example, these formats do not, by default, define explicit data types (e.g., string or float), which can lead to discrepancies during processing and larger resource requirements to infer data types "on the fly." Comma-delimited files are also difficult to compress in comparison to database formats (which are optimized to store data), and they are prone to errors such as quoting issues or manually typed errors, which can be nearly impossible to detect. Other formats, such as SQLite, provide explicit relationships between tables as an advantage but persist in using an ambiguous typing system that can result in, for example, strings in floating point columns (leading to similar data inference challenges).

A persistent and under-addressed challenge in image-based profiling is the lack of robust data-integration pathways between different image-analysis software feature extractors (e.g., CellProfiler,[6,7] DeepProfiler,[8] and Molecular Devices IN Carta[9]) and image-based profilers (e.g., Pycytominer[10]) (Figure 1B). While feature-extractor software can extract thousands of features from images, they typically require additional processing to become interoperable with downstream profiling. Specifically, these extractors do not provide standardized data or interfaces to support the transformation of features into structures compatible with image-based profiling workflows. As a result, researchers are frequently left to implement custom, ad hoc "stitch" code which, for example, borrows and rewrites existing code to join multiple CSVs, harmonize metadata, or reconcile segmentation identifiers in order to take full advantage of image-based profiling software. This process is not only error

This is an open access article under the CC BY license (http://creativecommons.org/licenses/by/4.0/).

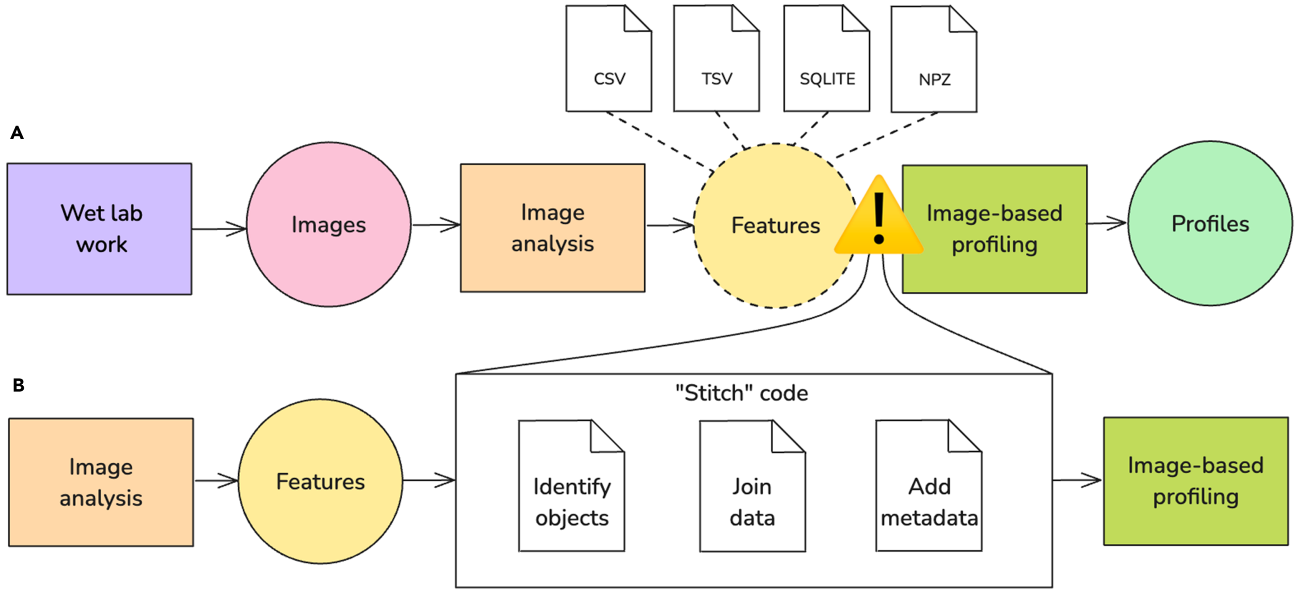

Figure 1. The standard data pipeline or bioinformatics workflow to analyze high-content imaging data

(A) The high-content imaging (HCI) pipeline starts with a hypothesis and wet lab work (a process symbolized by a rectangle) that results in the collection of microscopy images (data symbolized as a circle). Next, image analysis converts the images into morphology features, which then progress through an image-based profiling pipeline to generate processed profiles for downstream hypothesis testing and biological discovery. Image-analysis software produces features that are stored in myriad file formats from several different image-analysis tools. The output features from different image-analysis tools are inconsistent, posing several challenges (symbolized within the figure as a yield sign). For example, challenges include different naming conventions, numbers of files, and different data values.

(B) To solve these challenges, image analysts "stitch" code (rewrite existing and bring together previous code iterations) to integrate data prior to image-based profiling steps, which slows progress and may lead to reproducibility concerns.

prone but also inhibits reproducibility and scalability across experiments and laboratories by consuming valuable research time. Addressing this integration bottleneck is critical for enabling more automated, scalable, and reproducible HCI pipelines.

METHODS

CytoTable for feature integration

The increasing complexity and scale of HCI datasets demand computational tools that support scalable, interoperable, and reproducible analyses. The term "data integration" has been used to describe the technical challenge of combining heterogeneous biological datasets,[11] while more recent work emphasizes "data harmonization" as the process of reconciling differences in formats, definitions, and measurements across studies.[12] CytoTable addresses both needs by integrating diverse image-based feature-extraction outputs into a unified structure and harmonizing them into consistent, interoperable formats (e.g., Arrow,[13] Parquet,[14] and AnnData[15]) suitable for reproducible downstream analysis.[16] We developed CytoTable to bridge this gap as a scalable and reproducible "feature integrator" across many different image-based feature extractors and data formats (Figure 2A). CytoTable facilitates the transformation of raw image-based feature outputs into harmonized, bioinformatics-ready data by resolving complex multi-table relationships and enabling high-performance, single-cell data integration for downstream profiling (Figure 2B).

CytoTable applies the data-integration phase of the well-established data-processing and data-mining framework CRISP-DM.[17] By translating the heterogeneous outputs of individual feature extractors into a single, schema-driven table before any downstream analysis, it removes the tool-specific quirks that usually splinter HCI workflows and limit cross-study reuse. This standardized layer turns CytoTable from a mere extract, transform, and load utility into a reproducible, modular backbone on which image-based profiling pipelines can scale confidently and interoperably (Note S1).

CytoTable components

Inspired by modular data engineering principles and the Composable Data Management System Manifesto,[18] we designed CytoTable with a robust framework for managing varied image-analysis output files from HCI experiments. By leveraging a unified data representation, optimized query execution, and seamless cross-language interoperability, CytoTable enables researchers to efficiently process and analyze large-scale HCI data.

This conceptual model is grounded in long-term sustainable software practices[19,20] designed to promote maintainability alongside adaptability to avoid software collapse.[21] To meet this evolving landscape, we developed CytoTable using modular, interoperable components that reflect a commitment to modular and emergent design principles, inspired by the work of the Software Gardening Almanack.[22,23] The approach

CellPress
OPEN ACCESS

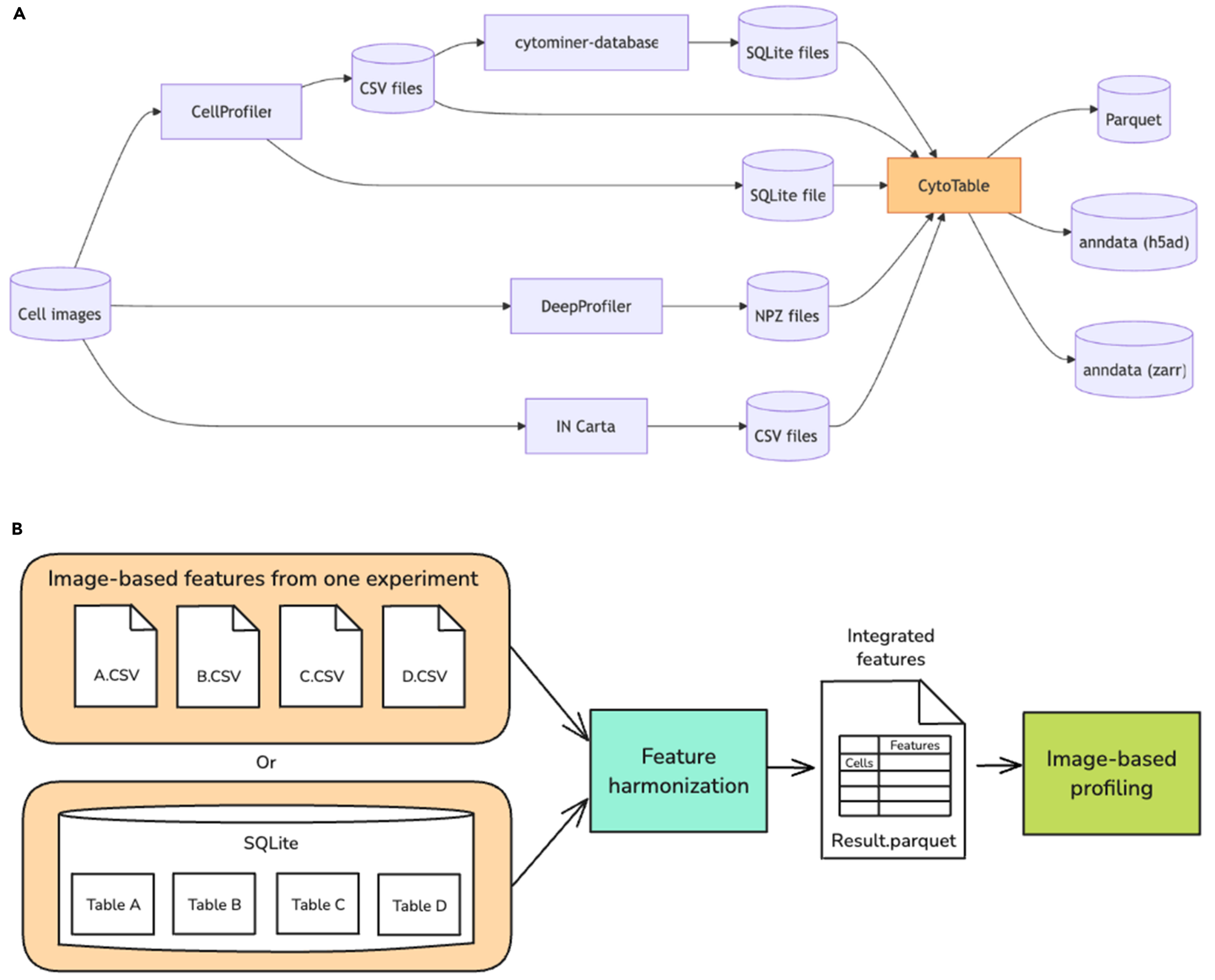

Figure 2. CytoTable solves the important problems of feature harmonization for image-based profiling

(A) CytoTable harmonizes a range of data formats commonly used in high-content imaging (HCI) workflows. These data sources originate from different image-analysis software tools widely adopted in the HCI community. Each output format is compatible with Pycytominer and other image-based profiling software.

(B) Through harmonization, CytoTable outputs file formats that integrate into downstream image-based profiling bioinformatics software. Feature harmonization through CytoTable involves consistent column name handling, data typing, and serialization to a number of formats.

not only increases the longevity of CytoTable but also leaves a durable foundation for future software tools and evolving standards in the image-based profiling community.

Cross-platform in-memory data types with Apache Arrow

At its core, CytoTable employs Apache Arrow[13] tables through PyArrow (the Pythonic API for Apache Arrow) for an intermediate in-memory representation, ensuring efficient columnar data access and minimizing the time it takes to prepare data within software procedures (Figure 3A). PyArrow tables within CytoTable handle all single-cell features to optimize processing performance and enforce strict typing expectations. Apache Arrow is an open-source, columnar in-memory data format designed to optimize data processing and interoperability across multiple programming languages. This structured approach allows users and maintainers to seamlessly transition between in-memory operations and long-term storage, facilitating reproducible workflows in image-based profiling.

Consistent and performant serialization through Parquet data storage

CytoTable leverages Apache Parquet[14] as its primary format for persistent data storage, ensuring efficient handling of large-scale single-cell HCI datasets. All intermediate and finalized feature data integrated by CytoTable are exported to Parquet. By adopting Parquet's columnar storage model, CytoTable significantly reduces disk I/O and improves query performance, particularly for analytical workloads that require scanning and aggregating large amounts of structured data. Its columnar layout is particularly advantageous for analytical queries, as it allows scanning only the necessary columns instead of entire rows, significantly improving query performance. Due to these advantages, Parquet is the foundation of

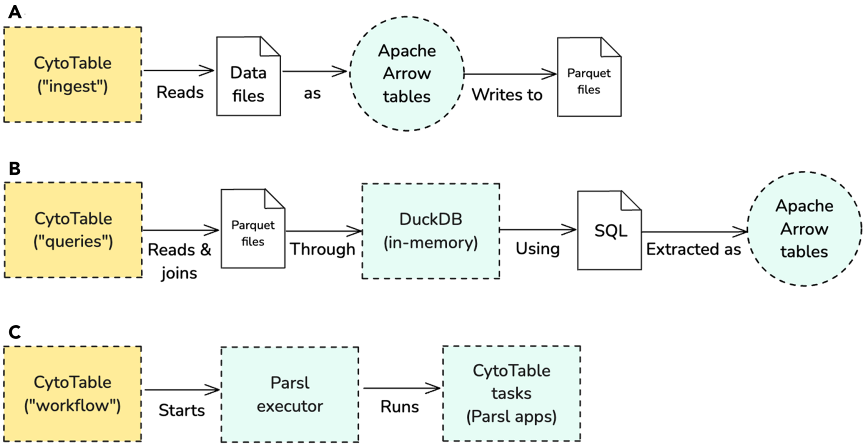

Figure 3. Core CytoTable processes

(A) CytoTable ingests serialized data from files as Apache Arrow tables using PyArrow. CytoTable then serializes the data files into Parquet files, retaining strong data-type relationships for Arrow integration.

(B) CytoTable reads and performs data joins through an in-memory DuckDB database as a query engine using SQL to extract Apache Arrow tables.

(C) CytoTable uses Parsl to orchestrate parallelizable tasks as a data workflow.

some of the largest data infrastructures in the world, including those used by Google BigQuery,[24] Amazon Athena,[25] Snowflake,[26] and Apache Spark.[27] Parquet's compression and encoding capabilities further enhance storage efficiency, making CytoTable well suited for workflows that involve iterative analysis and large-scale data sharing across computational environments.

Portable high-performance OLAP with DuckDB and SQL

To enhance cross-platform analytical performance, CytoTable integrates DuckDB,[28] an embedded analytical database designed for high-speed structured query language (SQL)-based queries on datasets (Figure 3B). SQL, a domain-specific language introduced in 1974[29] for managing data in relational database systems, remains one of the most widely adopted standards for querying and analyzing structured data. Its enduring relevance, portability, and declarative nature made it a natural choice for integration within CytoTable. DuckDB is an embedded analytical database designed for online analytical processing (OLAP), offering high-speed execution of complex queries on structured data. Unlike traditional transactional databases, DuckDB is optimized for read-heavy workloads, leveraging vectorized query execution and automatic optimizations to efficiently handle large datasets. Its ability to execute SQL queries directly within local environments, without requiring a separate database server, makes it an ideal tool for scalable data-analysis workflows. Further, DuckDB enables direct querying of Parquet or CSV data stored in cloud object storage (e.g., S3, GCS, or Azure Blob), eliminating the need for local downloads and supporting analyses across both local and distributed environments (see Note S2 for more information on cloud-based data access). CytoTable performs all extraction and join work on single-cell feature data through DuckDB SQL, enabling users to perform complex data transformations and aggregations with minimal computational overhead, scaling even to large-scale datasets (Note S2).

Scalable task-based parallel workflow execution with Parsl

CytoTable leverages Parsl,[30] a parallel programming library for Python, to implement MapReduce-style[31] workflows and distributed data processing. Parsl enables researchers to define modular, task-based execution pipelines that can scale seamlessly from local machines to high-performance computing clusters (Figure 3C). By abstracting task execution into a flexible dependency graph, Parsl facilitates efficient parallel execution of data transformations, distributing SQL queries, filtering operations, and other computational tasks across multiple processing units. This data-driven execution model is rooted in early dataflow semantics introduced by Dennis and Van Horn, where tasks are triggered by the availability of their inputs rather than by a centralized control.[32] This structure supports both multiprocessing and multithreading by decoupling task scheduling from the limitations of sequential programming.

It is important to understand how Python interacts with CPU cores and threads to realize these dataflow benefits on today's hardware. Modern computation relies on CPU cores, which are independent compute engines housed within each CPU package. Threads are an operating-system abstraction that the scheduler maps onto those cores. In CPython versions below 3.13, every process contains a global interpreter lock (GIL), a mutex that permits only one thread at a time to execute Python byte-code within that process. CPython versions 3.13 and above enable optional and eventual non-default GIL.[33] As a result, common and current CPython CPU-bound workloads may achieve true parallelism only with multiprocessing, which launches multiple processes (each with its own GIL) that the operating system (OS) can run concurrently on different cores. Multithreading creates several threads inside a single process; although the OS may place them on separate cores, they still contend for the same GIL, so the approach mainly benefits I/O-bound tasks or native libraries that release the lock. CytoTable supports both multiprocessing and multithreading, orchestrating single-cell data integration through Parsl's execution engines.

Accessible, extensible, and interoperable Python interface

CytoTable provides feature-integration capabilities to image-based profiling researchers by providing well-documented application programming interfaces (APIs) that can be readily installed as a Python package (e.g., "pip install cytotable"). Once installed, the Python package is importable into Python modules or scripts to flexibly implement feature integration into image-based profiling pipelines where it makes sense. CytoTable takes advantage of features afforded by Python packaging frameworks for automated testing, quality assurance, deployment, document rendering, and other tasks to promote

Table 1. CytoTable provides quick configuration options through the use of "presets," which are supplied at runtime

Software	Software output format	CytoTable preset
CellProfiler	CSV	cellprofiler_csv
CellProfiler	SQLite	cellprofiler_sqlite
DeepProfiler	NPZ	deepprofiler
IN Carta	CSV	in-carta
cytominer-database	SQLite	cell-health-cellprofiler-to-cytominer-database

Presets allow users to take advantage of common data formats output by image-analysis software to save time and reach data output sooner. Presets may be completely or partially overridden with individual CytoTable argument values for customized implementations as needed.

long-term project sustainability in one of the most used programming languages.

CytoTable is highly customizable to accommodate and harmonize multiple input sources

To accommodate different data schemas and conventions, CytoTable supports both manually specified configuration arguments and ready-made presets, which are included at installation. The configuration arguments enable flexibility when it comes to data ingestion, processing, and exports. Configuration presets (e.g., "cellprofiler_csv" or "in-carta") allow users to apply prepared configuration arguments for CytoTable in common data-format expectations without handcrafting specific SQL queries or other customizations (Table 1). When presets do not suffice, users can override specific options directly via arguments (allowing for partial use of presets). Each preset is versioned with the image-analysis software, and they are included in software tests to ensure deterministic and accurate results. A special parameter, "joins," within these presets helps document the SQL-based relationship between tables, which are extracted from image-analysis software. These presets are helpful in using CytoTable for known data sources or adjusting to meet new configurations.

CytoTable supports image-based profiling outputs from CellProfiler, an open-source image-analysis software.[6,7] It is widely used for cell segmentation, image quality control, and morphology feature extraction. It is often used in high-throughput screening, which generates many terabytes of data and demands high compute costs.[34,35] CellProfiler produces image-analysis outputs in many different forms, including CSV and SQLite data (Table 1). CellProfiler exports in CSV or SQLite formats, based, respectively, on the ExportToSpreadsheet or ExportToDatabase modules. CytoTable accommodates either output format with the existing presets, "cellprofiler_csv" and "cellprofiler_sqlite." Additionally, cytominer-database is a deprecated tool that cleans and processes CellProfiler output CSV files. We created a preset specially designed for these data called "cell-health-cellprofiler-to-cytominer-database." Because of its deprecation, we expect that this preset will only support legacy CellProfiler output data, which scientists may want to reanalyze.

CytoTable also supports image-based profiling outputs from DeepProfiler, which is an open-source deep-learning pipeline developed by the Cytomining community to generate morphological profiles directly from images using pretrained deep-learning models.[8] DeepProfiler outputs single-cell feature matrices in .npz format, which is a compressed NumPy archive suitable for efficient handling of high-dimensional data arrays. CytoTable provides the "deepprofiler" preset to harmonize these data.

Lastly, CytoTable supports image-based profiling outputs from IN Carta, which is a commercial image-analysis software platform developed by Molecular Devices[9] to provide tools for cell segmentation, tracking, and feature extraction. IN Carta exports processed image-based features as CSV files. These files contain per-object or per-image measurements suitable for morphological profiling and downstream analytics. CytoTable provides the "in-carta" preset for use with this format. Importantly, customizing presets is possible through overrides and customization. We expect that as other image-analysis feature data formats are developed, we (or other community members) can quickly develop new customized presets.

CytoTable benchmarking with other platforms and tools

We benchmarked join operations and memory usage between CytoTable and Pycytominer on realistic single-cell profiling tasks. Pycytominer is a bioinformatics software for processing image-based profiling data that contains a utility class called SingleCells.[10] This class, which is much more limited in scope than CytoTable, harmonizes CellProfiler output data prior to bioinformatics processing. We ran each benchmark six times per file type to quantify performance fluctuations.[36] We benchmarked using a Linux desktop computer with 16 CPU cores and 64 GB of RAM. Because of the limited functionality in Pycytominer, benchmarking comparisons between Pycytominer and CytoTable could only be directly performed using SQLite datasets. Therefore, our CSV dataset benchmarking used the Pandas[37] Python library rather than Pycytominer directly. All CytoTable comparisons involved two different Parsl execution engines, HighThroughputExecutor and ThreadPoolExecutor, labeled "multiprocess" and "multithread," respectively. We used Parsl's MonitoringHub[38] to profile memory for the HighThroughputExecutor because it involves specialized process spawning, which otherwise may result in inaccurate memory profiles. We performed all other memory profiling using psutil,[39] a commonly used and widely accepted tool within the Python community. We strove to provide an honest, best-effort benchmarking of these technologies.[40]

RESULTS

CytoTable comparison benchmarks

As image-based profiling workflows grow in scale and complexity, efficient data handling becomes increasingly important. We developed CytoTable in response to practical limitations encountered in earlier workflows, which revolve around in-memory operations using Pandas[37] DataFrames. While Pandas provides a flexible interface for data manipulation, its performance can degrade significantly when handling large-scale datasets, particularly during join operations, where multiple tables must be merged based on shared keys. These joins often require entire tables to be loaded and sometimes duplicated into memory, resulting in substantial resource

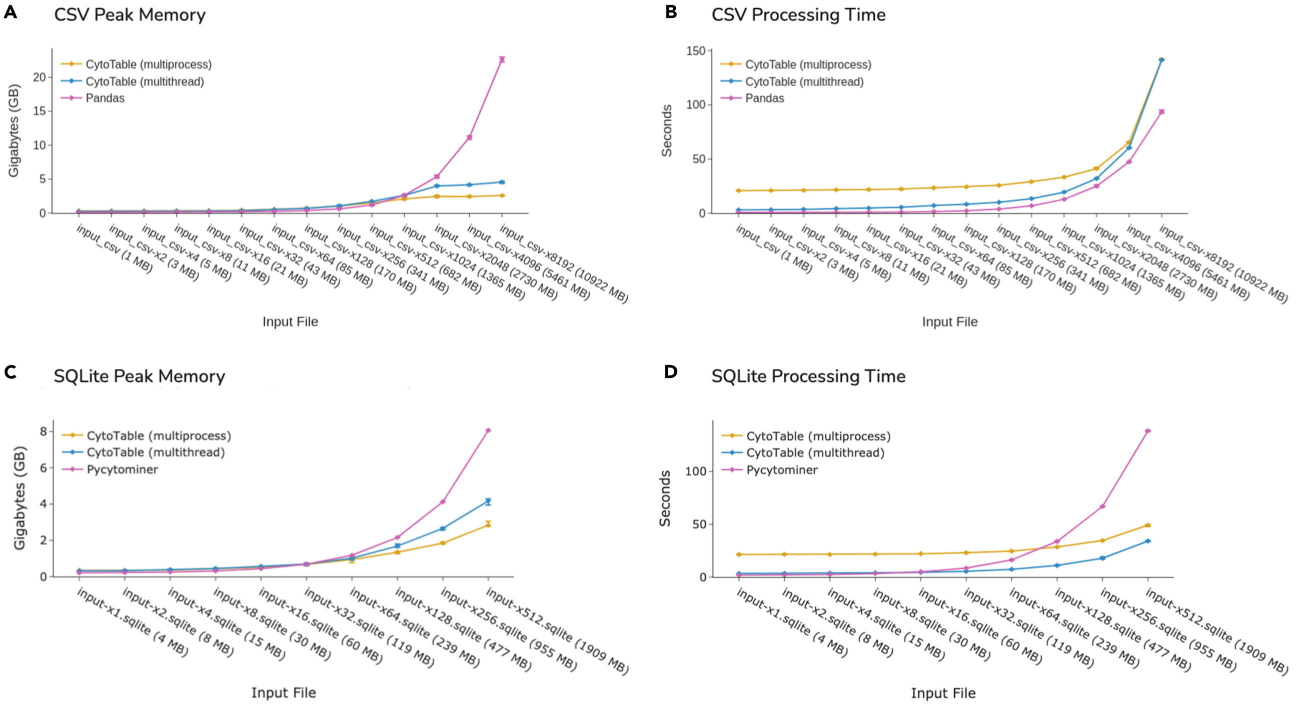

Figure 4. Benchmarking CytoTable and Pycytominer processing with increasingly large input data based on the NF1 Schwann Cell Project simulating larger data sizes with duplicated records. The error bars represent the full range of performance fluctuations for six independently executed runs per input file and tool.

(A) CytoTable outperforms Pandas for CSV-based memory performance.

(B) CytoTable outperforms Pandas in processing time for CSVs.

(C) CytoTable outperforms Pycytominer for SQLite memory performance.

(D) CytoTable outperforms Pycytominer in processing time for SQLite files.

consumption and potential scalability bottlenecks. A primary motivation for CytoTable was to reduce these memory requirements by leveraging more efficient data representations and execution strategies, enabling scalable data processing without compromising performance. We also developed CytoTable as a harmonization tool, which could accept multiple different image-analysis outputs. Pycytominer does not have this functionality, and we therefore wanted CytoTable to support newcomers (beyond CellProfiler users) into the Cytomining image-based profiling community.

When processing large CSVs, CytoTable uses several gigabytes less peak RAM than Pandas (Figure 4A), although Pandas still completes a few seconds faster (Figure 4B). For SQLite inputs, CytoTable again requires far less memory than Pycytominer (Figure 4C) and scales linearly, whereas Pycytominer's run time rises exponentially once file size exceeds 230 MB (Figure 4D). Within CytoTable itself, the default multiprocessing mode delivers the smallest memory footprint, while multithreading offers the shortest wall-clock time. Overall, Pandas is preferable for small CSV files, and Pycytominer's SingleCells.merge_single_cells method is preferable for small SQLite files, but CytoTable scales more gracefully as datasets grow, especially for SQLite files, providing a better speed-versus-memory trade-off. Ongoing work aims to further accelerate CSV parsing without sacrificing CytoTable's memory efficiency.

We chose to develop CytoTable using Apache Arrow, striking a balance between performance and flexibility. While tools like Pandas offer a user-friendly API and are widely adopted in data science, they can be inefficient when scaling to large datasets due to their reliance on dense in-memory structures. PyArrow serves as a common foundation across these ecosystems, offering a language-agnostic, columnar memory format that minimizes serialization costs and supports zero-copy data exchange. CytoTable adopts PyArrow Tables as its internal data representation to enable interoperability and standardize data types between file format and in-memory use. We observed that Apache Arrow tables through PyArrow or Polars[41] (another Arrow-compatible Python package) use roughly half the memory of Pandas DataFrames (Figure 5A).

Choosing the appropriate file format is critical for working with high-throughput image-based profiling data. CSV, though commonly used, lacks built-in support for types, compression, or metadata, which limits its suitability for large-scale structured data. SQLite improves on this by enabling queryable, relational storage in a single file, but it is not optimized for analytical workloads (table columns are by default limited to a maximum of 2,000 columns) and has limited parallel I/O capabilities. In contrast, CytoTable uses Apache Parquet by default for its persistent storage format. Parquet's columnar layout and native compression make it highly efficient for storing and retrieving subsets of large datasets, especially in scenarios involving

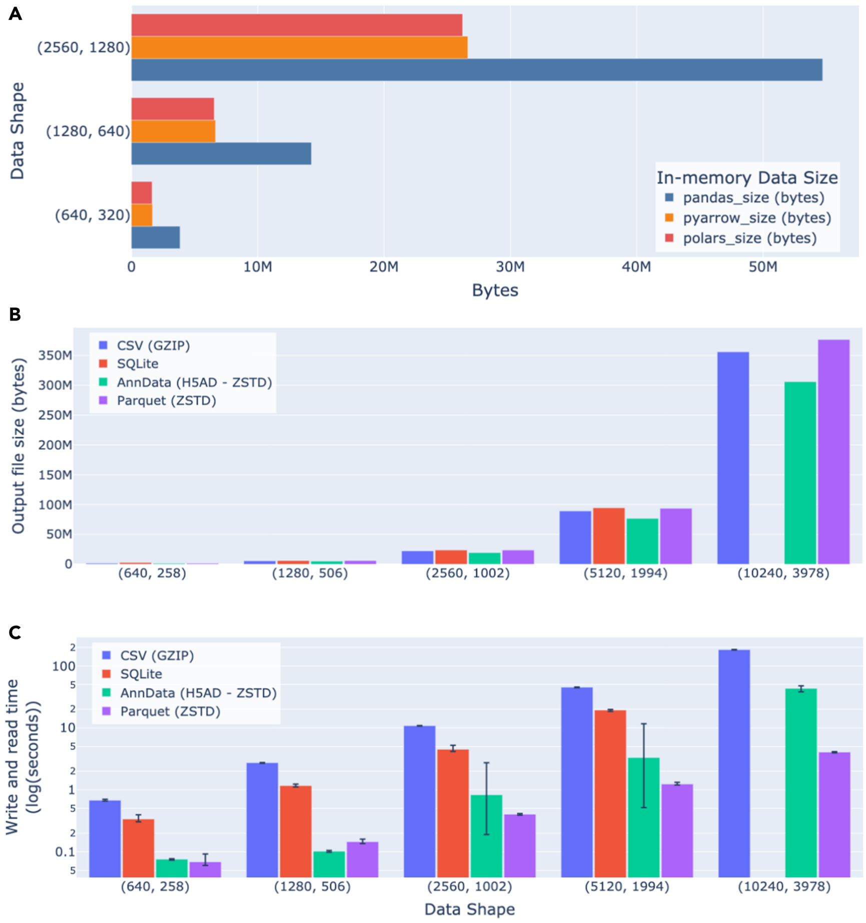

Figure 5. Benchmarking storage and file format performance

(A) Loading data through PyArrow tables uses almost half as much memory as Pandas DataFrames. We also compare Polars DataFrames, which leverage Arrow constructs to help contextualize how Arrow uses memory in different packages.

(B) Comparing output file sizes. AnnData achieves lower data storage when compared to CSV, SQLite, and Parquet. Parquet is equivalent in data-storage size or sometimes larger than CSV or SQLite files.

(C) Comparing write and read performance. Parquet consistently achieves better write- and read-time duration performance when compared with AnnData, SQLite, and CSV. Note that SQLite is incompatible for the largest data shape, given the default maximum column length of 2,000. Error bars represent the full range of six independent runs.

filtering or aggregation. Its compatibility with modern data systems and ability to handle high-throughput data-access patterns make it a natural choice for image-based profiling pipelines. We observed that Parquet may have roughly the same data storage size properties as SQLite or CSV files (Figure 5B) but outperforms both formats when it comes to read-time (Figure 5C) and write-time (Figure 5C) durations.

Beyond CSV and SQLite, AnnData (commonly used in the single-cell transcriptomics community) provides a rich container format for storing both data matrices and associated metadata. In benchmarking, we found that AnnData results in 17.5% smaller files on average than CSV, SQLite, or Parquet files (Figures 5B and 6). Parquet consistently outperforms AnnData in read and write times (up to 90% better, 34% average), even when both rely on identical compression algorithms (Figure 5C). In addition, Parquet supports partial writes and updates through multi-file dataset structures, which allow efficient appending or partitioning of large-scale data. Equivalent functionality in AnnData is currently only available through experimental projects such as AnnCollection. These practical advantages make Parquet a more scalable and flexible option for handling high-throughput image-based profiling data within CytoTable.

Applied use cases of CytoTable

To demonstrate CytoTable's practical utility across diverse experimental contexts, we highlight several applied use cases where it has already been integrated into real-world HCI workflows (Table 2). These examples span both small- and large-scale datasets, showcasing CytoTable's ability to standardize image-analysis outputs, support quality control, and streamline downstream analyses to enable reproducible, scalable data integration across a range of applications. These examples contextualize real-world projects with the benchmarks in the previous sections, illustrating that benchmarking expectations are mappable to applied use cases. Specifically, Figures 4 and 5 leveraged data from a single SQLite file from the NF1 Schwann Cell Project ranging from 242 to 123,904 single cells. Figure 6 used simulated datasets where each row represents a single cell (ranging from 640 to 10,240 simulated single cells).

Pyroptosis morphology map

Building on similar single-cell profiling workflows, Lippincott et al.[42] employed CytoTable to process data from over 8 million peripheral blood mononuclear cells subjected to various treatments to induce specific forms of cell death. Here, CytoTable was integrated into an image-based profiling pipeline alongside CellProfiler, facilitating the extraction and harmonization of nearly 3,000 features per cell. The formatted data enabled multimodal analyses linking morphology to inflammatory secretome responses, providing insights into mechanisms of programmed cell death. This effort involved one plate with 154 wells, 16 fields of view (FOVs), and around 8.3 million single cells (Table 2).

Pediatric cancer assay optimization

In an effort focused on optimizing experimental conditions for the Cell Painting assay applied across pediatric cancer cell lines, CytoTable was used to process morphological data.[43] Following segmentation and feature extraction by CellProfiler, CytoTable structured the single-cell profiles to support downstream processing with coSMicQC[44] and Pycytominer.[10] This

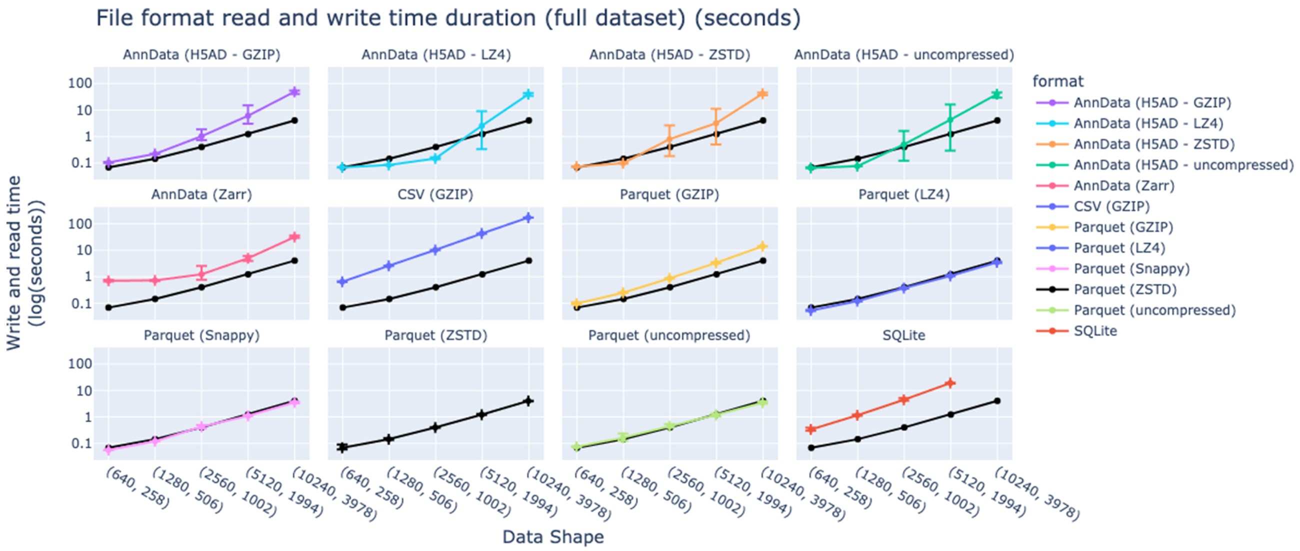

Figure 6. Benchmarking read and write time across data formats with varying compression options

Parquet has consistently better time performance for full dataset reads and writes (including through various compression algorithms) when compared to CSV, SQLite, and several AnnData formats. Note that SQLite is incompatible for the largest data shape, given the default maximum column length of 2,000. Error bars represent the full range of six independent runs.

formatting step helped evaluate how factors such as seeding density and assay timing influenced segmentation quality and morphological consistency, contributing to improved reproducibility in large-scale profiling workflows. This effort involved 24 plates with varying well counts and six total channels imaged (Table 2).

NF1 Schwann Cell Project

In the study by Tomkinson et al.,[45] researchers applied a modified Cell Painting assay to profile single-cell morphology in Schwann cells across different *NF1* genotypes. CytoTable was used to curate and format the high-dimensional segmentation outputs, enabling downstream integration with tools such as Pycytominer and coSMicQC. The resulting dataset of over 20,000 single-cell profiles supported quality control and statistical analyses, ultimately revealing subtle morphological signatures associated with *NF1* genotype. This effort involved four plates with varying well counts and four channels (Table 2).

Cytomining ecosystem project integration

Beyond individual studies, CytoTable plays a central role within the Cytomining ecosystem by providing standardized outputs that feed directly into downstream tools. Cytomining is a GitHub organization and community that develops and hosts image-based profiling bioinformatics software such as Pycytominer.[10] Pycytominer takes CytoTable data as input for image-based profiling workflows; coSMicQC uses CytoTable data to define thresholds for reliable quality control; and CytoDataFrame extends CytoTable output into interactive, in-memory exploration of single-cell profiles alongside images and segmentation masks. Together, these integrations demonstrate how CytoTable serves as a connective layer across Cytomining projects, enabling reproducible pipelines that span data harmonization, quality control, and exploratory analysis within the broader ecosystem of image-based profiling.

DISCUSSION

CytoTable is a core component of the Cytomining ecosystem, which is a vibrant open-source community focused on developing interoperable tools for HCI and high-throughput image-based profiling. The data outputs from CytoTable support streamlined input into downstream software such as coSMicQC,[44] which performs rigorous quality control on single-cell segmentation results, and Pycytominer,[10] a flexible framework for processing image-based features into formats compatible with statistical and machine-learning workflows. While each of these tools offers powerful capabilities on their own, they depend on a consistent, scalable data infrastructure to effectively bridge the gap between raw image-derived measurements from image-analysis tools and actionable biological insights. By delivering a standardized, memory-efficient, and

Table 2. CytoTable has been used in various research projects with varying data sizes to achieve image-based profiling harmonization

Use case	Plates	Wells	FOVs per well	Fluorescence channels	Estimated single cells
Pyroptosis morphology map	1	154	16	5	8.3 million
Pediatric cancer cell line assay optimization	39	7,134	9	6	9.2 million
NF1 Schwann Cell Project	4	184	20–35	4	24,000

Each project used customized versions of the CytoTable preset “cellprofiler_sqlite_pycytominer.”

SQL-compatible format for single-cell image-based profiling, CytoTable serves as a key integrative layer in this ecosystem.

As the image-based profiling field has matured through practical use and iterative development, several technical challenges and design considerations have emerged.[1] These considerations include in-memory and serialized data types, consistent floating-point numeric precision capture within in-memory data types, and memory allocator effectiveness combined with data types such as Apache Arrow (Note S3). These experiences have informed how CytoTable handles data format variability, numerical precision, and memory management across different execution environments. CytoTable solves these challenges for the image-based profiling field.

In the spirit of *panta rhei*—that everything flows and nothing stays the same—we acknowledge that the HCI and image-based profiling landscapes are constantly shifting. New tools, formats, and workflows continue to emerge, and any software aiming to support this community must evolve alongside it. CytoTable is built with this adaptability in mind: not as a rigid framework, but as a modular system designed to meet the changing needs of a diversity of bioimage informatics tasks. Looking ahead, we outline several key areas where CytoTable will continue to grow in response to this evolving ecosystem. CytoTable will continue to evolve as part of the broader Cytomining ecosystem, benefiting from shared standards, complementary tools, and an active open-source community. Iterative enhancements will focus on performance and scalability while maintaining strong interoperability with formats and workflows emerging across bioimaging and scverse[46] projects. By anchoring development within this collaborative ecosystem, CytoTable can adapt quickly to community needs and new image-analysis technologies, sustain reproducibility through shared testing and release practices, and provide a reliable foundation for advancing image-based profiling.

CytoTable is not without limitations. A current limitation is that its harmonization approach depends on morphology feature schema conventions and file formats that continue to evolve. As standards shift, sustained interoperability will require ongoing alignment and adaptation. While the open-source, ecosystem-driven model provides a pathway for addressing these challenges, it also means that reproducibility may be temporarily constrained if upstream tools or formats change faster than CytoTable can incorporate updates.

A central challenge in image-based profiling is the diversity of image-analysis formats, schemas, and workflows that make integration difficult.[47] CytoTable contributes toward a shared data model by harmonizing heterogeneous inputs into consistent, analysis-ready outputs, while remaining flexible enough to accommodate emerging tools and standards. Continued progress in this direction will reduce duplication, lower barriers to interoperability, and support the long-term goal of a collaboratively developed model for single-cell morphology data across multiple organizations (such as that of scverse).

Future development: Expanding input sources

While CytoTable currently provides robust support for standard image-based profiling outputs, future development will expand its compatibility with a broader range of image-analysis input types through additional presets and custom configurations. Currently tailored to formats such as CellProfiler's CSV and SQLite exports, upcoming versions aim to natively support additional tools and pipelines in the image-based profiling ecosystem. This expansion will improve interoperability across profiling tools and reduce the need for custom preprocessing scripts, making CytoTable a more flexible entry point into standardized single-cell morphology analysis workflows.

Future development: Enhancing output format options

Emerging formats such as Lance,[48] Nimble[49] (initially called "Alpha"), and Vortex[50] offer new opportunities for scalable data storage, streaming access, and versioned datasets optimized for analytical performance and cloud-native environments. Integrating these formats will provide users with greater flexibility in managing large-scale single-cell datasets, particularly in settings that demand fast I/O, partial reads, or remote data access. Lance in particular includes array-value capabilities, which are a gap of Parquet and could be leveraged to great effect with regard to features, image data (as arrays), or shape vectors (of image objects). It also is optimized for vector search, which could offer benefits to profiling efforts.

Future development: Integrating with CytoDataFrame

Finally, we plan a deeper integration with CytoDataFrame,[51] an in-memory data abstraction for single-cell image-based profiling. We designed CytoDataFrame to couple cell measurements with associated metadata, segmentation masks, and raw images in memory, supporting interactive analysis and visualization in environments such as Jupyter. CytoTable, in contrast, is focused on feature harmonization and serialization into file formats. Future work will explore bidirectional compatibility between CytoTable and CytoDataFrame (e.g., enabling .to_cytotable() and .from_cytotable() methods) to bridge persistent storage and live, human-in-the-loop exploration that is required for iterative development and hypothesis generation.

RESOURCE AVAILABILITY

Lead contact

Requests for further information and resources should be directed to and will be fulfilled by the lead contact, Gregory P. Way (gregory.way@cuanschutz.edu).

Materials availability

No new materials were generated in this study.

Data and code availability

- CytoTable is an open-source project, and its source code can be viewed and downloaded from https://github.com/cytomining/cytotable (https://doi.org/10.5281/ZENODO.14888111).
- CytoTable's installation and usage documentation is available at https://cytomining.github.io/CytoTable/.
- A brief tutorial on how to use CytoTable is available at https://cytomining.github.io/CytoTable/tutorial.html.
- A notebook-based example notebook showing CytoTable in use is available at https://cytomining.github.io/CytoTable/examples/cytotable_mise_en_place.html.
- A notebook-based example notebook showing CytoTable in use with cloud data is available at https://cytomining.github.io/CytoTable/examples/cytotable_from_the_cloud.html.
- The repository containing the code used to conduct benchmarking and generate results is available at https://github.com/cytomining/CytoTable-benchmarks (https://doi.org/10.5281/ZENODO.15425830).

ACKNOWLEDGMENTS

J.T., D.B., and G.P.W. were supported in part by Alex's Lemonade Stand Foundation "A" award and Tap Cancer Out (grant #23-28306 to G.P.W.). G.P.W. and E.S. were supported in part by an American Heart Association Collaborative Sciences Award (24CSA1255857) to G.P.W. Special thanks goes to the following for their help in contributing to CytoTable design and development or related work: Cameron Mattson from Way Lab (https://www.waysciencelab.com/); Shantanu Singh, Beth Cimini, and Sam Chen from Broad Institute (https://www.broadinstitute.org/); Samir Amin from Yale School of Medicine (https://medicine.yale.edu/); Juan C. Caicedo and Nikita Moshkov from University of Wisconsin-Madison Morgridge Institute for Research (https://morgridge.org/); and Alexandre Colas and Aashna Lamba from Sanford Burnham Prebys (https://sbpdiscovery.org/).

AUTHOR CONTRIBUTIONS

Conceptualization, D.B. and G.P.W.; data curation, D.B., J.T., and E.S.; formal analysis, D.B. and J.T.; funding acquisition, G.P.W.; investigation, D.B., J.T., E.S., M.J.L., K.I.B., and G.P.W.; methodology, D.B., J.T., E.S., M.J.L., K.I.B., F.A., and G.P.W.; project administration, D.B. and G.P.W.; resources, V.R. and G.P.W.; software, D.B., J.T., E.S., M.J.L., K.I.B., F.A., and G.P.W.; supervision, D.B. and G.P.W.; validation, D.B. and F.A.; visualization, D.B.; writing – original draft, D.B. and G.P.W.; writing – review & editing, D.B., J.T., E.S., M.J.L., K.I.B., V.R., F.A., and G.P.W.

DECLARATION OF INTERESTS

K.I.B. is an employee of Seqera Labs S.L.

DECLARATION OF GENERATIVE AI AND AI-ASSISTED TECHNOLOGIES IN THE WRITING PROCESS

During the preparation of this work the authors used OpenAI's ChatGPT in order to improve the readability and language of the manuscript. After using this tool/service, the authors reviewed and edited the content as needed and take full responsibility for the content of the published article.

SUPPLEMENTAL INFORMATION

Supplemental information can be found online at https://doi.org/10.1016/j.patter.2026.101514.

Received: June 19, 2025
Revised: October 16, 2025
Accepted: February 23, 2026
Published: March 30, 2026

REFERENCES

1. Caicedo, J.C., Cooper, S., Heigwer, F., Warchal, S., Qiu, P., Molnar, C., Vasilevich, A.S., Barry, J.D., Bansal, H.S., Kraus, O., et al. (2017). Data-analysis strategies for image-based cell profiling. Nat. Methods *14*, 849–863.
2. Seal, S., Trapotsi, M.-A., Spjuth, O., Singh, S., Carreras-Puigvert, J., Greene, N., Bender, A., and Carpenter, A.E. (2025). Cell Painting: a decade of discovery and innovation in cellular imaging. Nat. Methods *22*, 254–268.
3. Way, G.P., Sailem, H., Shave, S., Kasprowicz, R., and Carragher, N.O. (2023). Evolution and impact of high content imaging. SLAS Discov. *28*, 292–305.
4. Cimini, B.A., Bankhead, P., D'Antuono, R., Fazeli, E., Fernandez-Rodriguez, J., Fuster-Barceló, C., Haase, R., Jambor, H.K., Jones, M.L., Jug, F., et al. (2024). The crucial role of bioimage analysts in scientific research and publication. J. Cell Sci. *137*, jcs262322.
5. Cohen, J., Katz, D.S., Barker, M., Chue Hong, N., Haines, R., and Jay, C. (2021). The four pillars of research software engineering. IEEE Softw. *38*, 97–105.
6. Carpenter, A.E., Jones, T.R., Lamprecht, M.R., Clarke, C., Kang, I.H., Friman, O., Guertin, D.A., Chang, J.H., Lindquist, R.A., Moffat, J., et al. (2006). CellProfiler: image analysis software for identifying and quantifying cell phenotypes. Genome Biol. *7*, R100.
7. Stirling, D.R., Swain-Bowden, M.J., Lucas, A.M., Carpenter, A.E., Cimini, B.A., and Goodman, A. (2021). CellProfiler 4: improvements in speed, utility and usability. BMC Bioinf. *22*, 433.
8. Moshkov, N., Bornholdt, M., Benoit, S., Smith, M., McQuin, C., Goodman, A., Senft, R.A., Han, Y., Babadi, M., Horvath, P., et al. (2024). Learning representations for image-based profiling of perturbations. Nat. Commun. *15*, 1594.
9. Molecular Devices. IN Carta: Image Analysis Software for High-Content Screening. https://www.moleculardevices.com/products/cellular-imaging-systems/high-content-analysis/in-carta-image-analysis-software.
10. Serrano, E., Chandrasekaran, S.N., Bunten, D., Brewer, K.I., Tomkinson, J., Kern, R., Bornholdt, M., Fleming, S.J., Pei, R., Arevalo, J., et al. (2025). Reproducible image-based profiling with Pycytominer. Nat. Methods *22*, 677–680.
11. Lapatas, V., Stefanidakis, M., Jimenez, R.C., Via, A., and Schneider, M.V. (2015). Data integration in biological research: an overview. J. Biol. Res. (Thessalon.) *22*, 9.
12. Cheng, C., Messerschmidt, L., Bravo, I., Waldbauer, M., Bhavikatti, R., Schenk, C., Grujic, V., Model, T., Kubinec, R., and Barceló, J. (2024). A general primer for data harmonization. Sci. Data *11*, 152.
13. Apache Software Foundation. Apache Arrow: A Cross-Language Development Platform for In-Memory Analytics. https://arrow.apache.org/.
14. Apache Software Foundation. Apache Parquet: Columnar Storage Format. https://parquet.apache.org/.
15. Virshup, I., Rybakov, S., Theis, F.J., Angerer, P., and Wolf, F.A. (2024). anndata: Access and store annotated data matrices. J. Open Source Softw. *9*, 4371.
16. Bunten, D., Tomkinson, J., Serrano, E., Lippincott, M., Brewer, K., Rubinetti, V., Alquaddoomi, F., and Way, G. (2025). CytoTable (Zenodo). https://doi.org/10.5281/zenodo.14888111.
17. Shearer, C., and The, C.-D. (2000). The CRISP-DM Model: The New Blueprint for Data Mining. J. Data Warehous *5*, 13–22.
18. Pedreira, P., Erling, O., Karanasos, K., Schneider, S., McKinney, W., Valluri, S.R., Zait, M., and Nadeau, J. (2023). The Composable Data Management System Manifesto. Proc. VLDB Endow *16*, 2679–2685.
19. Wilson, G., Aruliah, D.A., Brown, C.T., Chue Hong, N.P., Davis, M., Guy, R.T., Haddock, S.H.D., Huff, K.D., Mitchell, I.M., Plumbley, M.D., et al. (2014). Best practices for scientific computing. PLoS Biol. *12*, e1001745.
20. Wilson, G., Bryan, J., Cranston, K., Kitzes, J., Nederbragt, L., and Teal, T.K. (2017). Good enough practices in scientific computing. PLoS Comput. Biol. *13*, e1005510.
21. Hinsen, K. (2019). Dealing with software collapse. Comput. Sci. Eng. *21*, 104–108.
22. Bunten, D., Davidson, W., and Way, G.P. Growing resilient scientific software ecosystems: Introducing the Software Gardening Almanack. Better Scientific Software (BSSw) blog. https://bssw.io/blog_posts/growing-resilient-scientific-software-ecosystems-introducing-the-software-gardening-almanack.
23. Bunten, D., Davidson, W., Alquaddoomi, F., Rubinetti, V., and Way, G. (2025). The Software Gardening Almanack (Zenodo). https://doi.org/10.5281/zenodo.14765834.
24. Melnik, S., Gubarev, A., Long, J.J., Romer, G., Shivakumar, S., Tolton, M., and Vassilakis, T. (2010). Dremel: interactive analysis of web-scale datasets. Proceedings VLDB Endowment *3*, 330–339.
25. Amazon Web Services. Amazon Athena. https://aws.amazon.com/athena/.
26. Dageville, B., Cruanes, T., Zukowski, M., Antonov, V., Avanes, A., Bock, J., Claybaugh, J., Engovatov, D., Hentschel, M., Huang, J., et al. (2016). The snowflake elastic data warehouse. In Proceedings of the 2016

International Conference on Management of Data (ACM). https://doi.org/10.1145/2882903.2903741.

27. Zaharia, M. (2012). Resilient distributed datasets: a fault-tolerant abstraction for in-memory cluster computing. In Proceedings of the 9th USENIX Conference on Networked Systems Design and Implementation Association.
28. Raasveldt, M., and Mühleisen, H. (2019). DuckDB. In Proceedings of the 2019 International Conference on Management of Data (ACM), pp. 1981–1984.
29. Chamberlin, D.D. (2012). Early History of SQL. IEEE Ann. Hist. Comput. *34*, 78–82.
30. Babuji, Y., Woodard, A., Li, Z., Katz, D.S., Clifford, B., Kumar, R., Lacinski, L., Chard, R., Wozniak, J.M., Foster, I., et al. (2019). Parsl. In Proceedings of the 28th International Symposium on High-Performance Parallel and Distributed Computing (ACM).
31. Dean, J., and Ghemawat, S. (2008). MapReduce: simplified data processing on large clusters. Commun. ACM *51*, 107–113.
32. Dennis, J.B., and Van Horn, E.C. (1983). Programming semantics for multiprogrammed computations. Commun. ACM *26*, 29–35.
33. PEP 703 – making the global interpreter lock optional in CPython Python Enhancement Proposals (PEPs). https://peps.python.org/pep-0703/.
34. Way, G.P., Natoli, T., Adeboye, A., Litichevskiy, L., Yang, A., Lu, X., Caicedo, J.C., Cimini, B.A., Karhohs, K., Logan, D.J., et al. (2022). Morphology and gene expression profiling provide complementary information for mapping cell state. Cell Syst. *13*, 911–923.e9.
35. Chandrasekaran, S.N., Cimini, B.A., Goodale, A., Miller, L., Kost-Alimova, M., Jamali, N., Doench, J.G., Fritchman, B., Skepner, A., Melanson, M., et al. (2024). Three million images and morphological profiles of cells treated with matched chemical and genetic perturbations. Nat. Methods *21*, 1114–1121.
36. Bunten, D., Lippincott, M., Alquaddoomi, F., and Way, G. (2025). CytoTable-benchmarks (Zenodo). https://doi.org/10.5281/zenodo.15425830.
37. McKinney, W. (2010). Data Structures for Statistical Computing in Python. In Proceedings of the Python in Science Conference (SciPy), pp. 56–61.
38. Monitoring — Parsl 1.3.0-dev documentation. https://parsl.readthedocs.io/en/stable/userguide/advanced/monitoring.html.
39. Rodola, G. psutil: Cross-platform lib for process and system monitoring in Python (GitHub).https://psutil.readthedocs.io/en/latest/
40. Raasveldt, M., Holanda, P., Gubner, T., and Mühleisen, H. (2018). Fair benchmarking considered difficult. In Proceedings of the Workshop on Testing Database Systems (ACM), pp. 1–6.
41. Polars - DataFrames for the new era. https://pola.rs/.
42. Lippincott, M.J., Tomkinson, J., Bunten, D., Mohammadi, M., Kastl, J., Knop, J., Schwandner, R., Huang, J., Ongo, G., Robichaud, N., et al. (2025). A morphology and secretome map of pyroptosis. Mol. Biol. Cell. *36*, https://doi.org/10.1091/mbc.E25-03-0119.
43. Tomkinson, J., Serrano, E., Curd, J., Li, W., and Way, G. (2025). pediatric_cancer_atlas_profiling (Zenodo). https://doi.org/10.5281/zenodo.15548848.
44. Tomkinson, J., Bunten, D., and Way, G.P. (2025). Stellar quality control for single-cell image-based profiling with coSMicQC. Preprint at bioRxiv. https://doi.org/10.1101/2025.10.14.682427.
45. Tomkinson, J., Mattson, C., Mattson-Hoss, M., Guzman, G., Sarnoff, H., Bouley, S.J., Walker, J.A., and Way, G.P. (2025). High-content microscopy and machine learning characterize a cell morphology signature of NF1 genotype in Schwann cells. Glial Health Research *2*, 100009.
46. Virshup, I., Bredikhin, D., Heumos, L., Palla, G., Sturm, G., Gayoso, A., Kats, I., Koutrouli, M., Scverse Community, Berger, B., et al. (2023). The scverse project provides a computational ecosystem for single-cell omics data analysis. Nat. Biotechnol. *41*, 604–606.
47. Serrano, E., Peters, J., Wagner, J., Graham, R.E., Chen, Z., Feng, B., Miranda, G., Kalinin, A.A., Vulliard, L., Tomkinson, J., et al. (2025). Progress and new challenges in image-based profiling. Preprint at arXiv. https://doi.org/10.48550/arXiv.2508.05800.
48. Pace, W., She, C., Xu, L., Jones, W., Lockett, A., Wang, J., and Shah, R. (2025). Lance: Efficient random access in columnar storage through adaptive structural encodings. Preprint at arXiv. https://doi.org/10.48550/ARXIV.2504.15247.
49. Chattopadhyay, B. (2023). Shared Foundations: Modernizing Meta's Data Lakehouse. In 13th Conference on Innovative Data Systems Research, CIDR 2023.
50. Vortex – LFAI & data. https://lfaidata.foundation/projects/vortex/.
51. Bunten, D., Tomkinson, J., Rubinetti, V., and Way, G. (2025). CytoDataFrame (Zenodo). https://doi.org/10.5281/ZENODO.14797074.

Patterns, Volume 7

Supplemental information

Scalable data harmonization for single-cell image-based profiling with CytoTable

Dave Bunten, Jenna Tomkinson, Erik Serrano, Michael J. Lippincott, Kenneth I. Brewer, Vince Rubinetti, Faisal Alquaddoomi, and Gregory P. Way

Supplemental Note 1 - CytoTable Framework Philosophy
By framing high-content imaging (HCI) explicitly within data mining methodologies, we can better modularize image-based profiling steps, ensure interoperability, and support reproducibility. This modular perspective not only appreciates the strengths of existing tools but also encourages broader collaboration across the open-source ecosystem, ensuring that HCI evolves as a cohesive and community-driven scientific practice.

Within the CRISP-DM (Cross-Industry Standard Process for Data Mining) framework, data integration is recognized as a foundational stage in preparing datasets for analysis [S1]. Positioned between data understanding and modeling, this phase ensures that disparate sources, which are often heterogeneous in format and schema, are transformed into a coherent structure suitable for downstream interpretation and modeling. In the context of HCI, where multiple outputs from segmentation pipelines, metadata annotations, and image-derived features must be reconciled, robust data integration is especially critical. More broadly, the lines between data mining and data science blur: while data science emphasizes exploration, hypothesis generation, and flexible analytical tooling, data mining traditionally focuses on producing structured, repeatable outputs such as predictions, classifications, or segmentations through well-defined pipelines [S2]. Effective data integration lies at the heart of both, serving as the bridge between raw inputs and actionable insights. As such, tools like CytoTable play an essential role in operationalizing this step, enabling both scientific exploration and reliable downstream application.

Supplemental Note 2 - CytoTable data methods
CytoTable is a modular, scalable data processing library designed for transforming and managing image-derived, high-content, single-cell data. To accommodate diverse workflows and ensure flexibility across computing environments, CytoTable integrates task-based execution via Parsl. Below, we describe the architecture of CytoTable workflows and how they integrate cloud-native data access, efficient in-memory operations, and flexible configuration mechanisms.

CytoTable pagination
Working with large datasets can pose significant challenges, particularly in environments with constrained memory resources that cannot accommodate entire tables in memory simultaneously. For instance, when loading a full table with Pandas [S3], the system must allocate enough memory to hold the entire dataset. If the system doesn't have enough memory, Python may crash or encounter out-of-memory errors. CytoTable addresses this limitation through data pagination, a method that processes data in smaller, manageable subsets (often referred to as "chunks") to avoid memory bottlenecks (**Figure S1**). CytoTable controls pagination behavior by two parameters: "chunk_size", which defines the maximum number of rows per page, and "page_keys", which specify the column(s) used to partition the data. Using these settings, CytoTable dynamically generates data pages and exports each as an individual Parquet file. Together, these files form a larger Parquet "dataset," enabling scalable and memory-efficient table processing.

Original

"Data source"

Col_A	Col_B	Col_C
1	a	0.01
2	b	0.02

Changes

Page or "Chunk" 1

Col_A	Col_B	Col_C
1	a	0.01

Page or "Chunk" 2

Col_A	Col_B	Col_C
2	b	0.02

Figure S1. *CytoTable paginates data sources into pages or "chunks", which are sets of records from the data source, to help reduce memory strain and increase scalable performance.*

CytoTable data concatenation and joins

CytoTable supports two core methods for reconstructing and integrating data across paginated or distributed sources: concatenation and joins. Concatenation refers to the vertical stacking of multiple data "chunks" that share identical column schemas, effectively reassembling a full dataset from previously partitioned subsets (**Figure S2**). This process reverses the pagination operation and is implemented using a sequential Parquet writer to combine multiple Parquet files into a unified dataset without loading the entirety of the concatenated dataset.

Original

Page or "Chunk" 1

Col_A	Col_B	Col_C
1	a	0.01

Page or "Chunk" 2

Col_A	Col_B	Col_C
2	b	0.02

Changes

"Concatenated data"

Col_A	Col_B	Col_C
1	a	0.01
2	b	0.02

Figure S2. *Concatenation of pages of tabular data with the same columns into singular tables.*

In contrast, joins enable horizontal merging of datasets with overlapping key columns but differing auxiliary data (e.g., metadata)(**Figure S3**). These join operations are expressed using DuckDB SQL syntax within CytoTable's convert() function, allowing users to declaratively specify SQL-style semantics such as LEFT JOIN or INNER JOIN. While some tools use the term "merge" for similar operations (e.g., pandas.DataFrame.merge()), CytoTable explicitly adopts SQL-style "join" terminology to maintain clarity and consistency with database engines like DuckDB, which it leverages under the hood. We use this methodology because data joins through the Pythonic API of DataFrame libraries such as Pandas, Polars, Dask, or Modin all differ in parameter or operation making it otherwise more difficult to discern consistency.

Original	Changes

"Table 1" (notice **Col_C**)

Col_A	Col_B	Col_C
1	a	0.01

"Table 2" (notice **Col_Z**)

Col_A	Col_B	Col_Z
1	a	2024-01-01

"Joined data" (Table 1 left-joined with Table 2)

Col_A	Col_B	Col_C	Col_Z
1	a	0.01	2024-01-01

Join Specification in SQL

```sql
SELECT *
FROM Table_1
LEFT JOIN Table_2 ON
Table_1.Col_A = Table_2.Col_A;
```

Figure S3. *Data joins allow for data table sources with joinable keys but disparate columns to be brought together as a new dataset of mixed columns based on the specification of the join (for example, using SQL).*

Relational joins are central to many bioimaging workflows, especially when combining metadata with single-cell measurements. While Pandas' merge() function is commonly used, it requires full in-memory loading of datasets and can be resource-intensive. SQLite supports basic joins, but lacks the performance optimizations necessary for large-scale analytical queries. CytoTable instead relies on DuckDB, an embedded analytical SQL engine designed for efficient local execution on structured data. DuckDB executes joins using a vectorized query engine and operates directly on Arrow and Parquet data, eliminating unnecessary serialization and allowing for performant, scalable data integration. This makes it especially well-suited for workflows that involve complex filtering, aggregation, or cross-table joins in resource-constrained environments. Through benchmarking we observed that DuckDB outperforms Pandas for time duration and memory consumption with larger files, whereas Pandas outperforms DuckDB with smaller data (**Figure S4**).

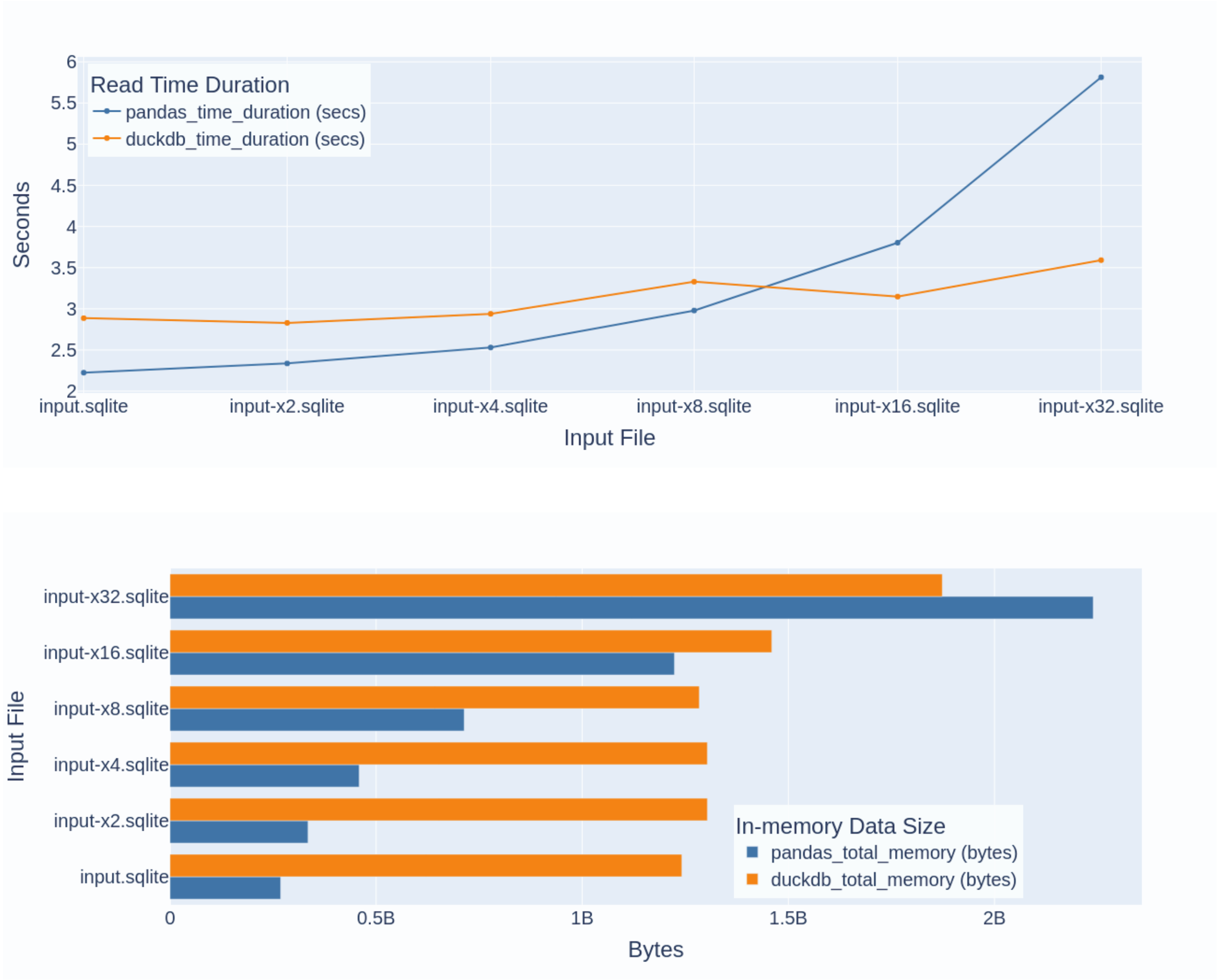

Figure S4. *Performance benchmarks of DuckDB and Pandas.* (Top panel) DuckDB required slightly more time than Pandas for join operations, but it surpassed Pandas in speed once input size exceeded ~30 MB (SQLite benchmark). (Bottom panel) DuckDB consistently used less memory than Pandas across larger datasets.

CytoTable MapReduce

CytoTable adopts a MapReduce paradigm to enable scalable and modular processing of large datasets. In the "map" phase, CytoTable processes data at the level of individual pages, allowing transformations, computations, or annotations to be applied independently and in parallel (**Figure S5**). This design not only optimizes memory usage but also lends itself naturally to distributed or batch processing frameworks. In the subsequent "reduce" phase, CytoTable recombines the processed pages into unified data structures. For example, pages may be concatenated to reconstruct the full table or joined with metadata and annotations to create enriched, analysis-ready datasets. This flexible approach enables CytoTable to handle large-scale data workflows efficiently.

Original	Paginated	Changed	Reduced

Original

“Data source”

Col_A	Col_B	Col_C
1	a	0.01
2	b	0.02

Paginated

Page or “Chunk” 1

Col_A	Col_B	Col_C
1	a	0.01

Page or “Chunk” 2

Col_A	Col_B	Col_C
2	b	0.02

Changed

Modified Page or “Chunk” 1

Table_ID	Col_A	Col_B	Col_C
123	1	a	0.01

Modified Page or “Chunk” 2

Table_ID	Col_A	Col_B	Col_C
123	2	b	0.02

Reduced

“Reduced” data result (concatenated)

Table_ID	Col_A	Col_B	Col_C
123	1	a	0.01
123	2	b	0.02

Figure S5. *MapReduce techniques enable mapped changes to take place on paginated sets which can be reduced to data summaries as concatenated tables.*

CytoTable has unified path handling for local or cloud-based data

CytoTable supports both local and remote data sources through a unified interface powered by the cloudpathlib library (**Figure S6**). This allows users to process data stored on Amazon S3, Google Cloud Storage, and Azure Blob Storage as if they were local files (leveraging commonly used paths denoted such as “s3://bucket/file”). For example, CellProfiler outputs hosted on public-facing S3 buckets can be read directly without authentication (for example, by leveraging “no-sign-request” configuration options).

For cloud-based SQLite sources, which require local access due to SQLite’s file system constraints, CytoTable uses cloudpathlib’s caching capabilities to download the database prior to processing. To avoid issues with system memory constraints in temporary directories, users may specify a custom cache directory (local_cache_dir). This balances the ability to use cloud-based paths even for data which may not inherently be streamable.

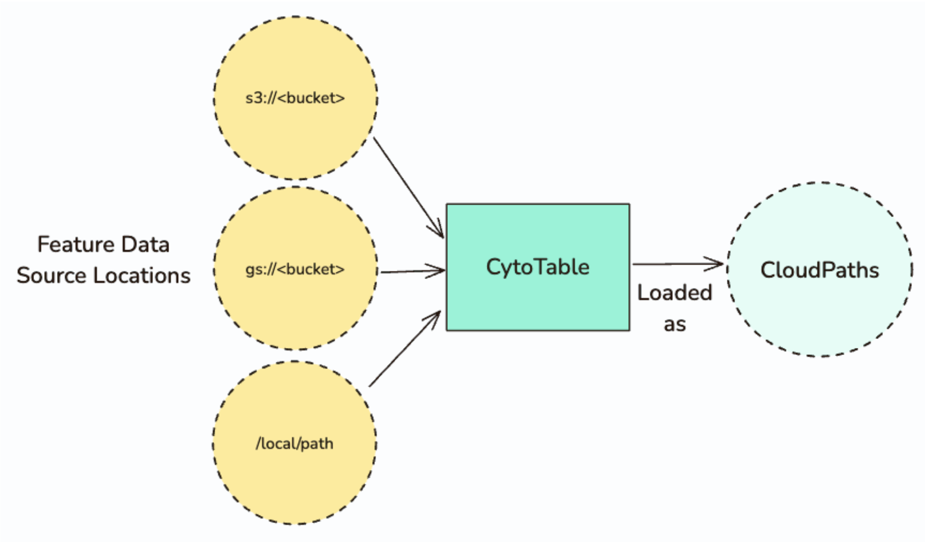

Figure S6. *Local paths and cloud-based paths can be handled as abstract CloudPaths within CytoTable making it easy for users to leverage datasets from many different locations without changing their code using recognizable filesystem patterns. Without CytoTable and CloudPaths, scientific developers would need to implement more custom “stitch” code to stream or download cloud-based data so it may be treated as locally available data.*

Supplemental Note 3 - CytoTable solves core image-based profiling challenges

SQLite type flexibility challenges

CytoTable uses SQLite as a source database format that is converted into more structured representations for downstream analysis. One of SQLite's distinguishing characteristics is its use of flexible typing, a system where column types are treated as type affinities rather than strict constraints. In this model, SQLite applies a best-effort mapping between the declared column type and the actual storage class of each value. In other words, values in a single column can legally belong to different internal storage types [S4]. For example, a column declared as REAL may contain both 0.01 (a floating-point number) and "value" (a string), corresponding to the REAL and TEXT storage classes, respectively. This flexibility can lead to challenges when consuming such data with systems that expect uniform data types.

Additionally, SQLite supports constraints like NOT NULL, which explicitly prevent the use of the NULL storage class, SQLite's marker for missing values. In practice, we encountered source tables where columns were constrained with NOT NULL but contained values such as "nan"—a string literal we inferred to represent numpy.nan [S5], a NumPy standard in Python for null-like values in floating-point arrays. This kind of inconsistency is tolerated by SQLite, but creates friction when interoperability with strictly typed systems is required. In contrast, engines like DuckDB, as well as libraries like Apache Arrow and NumPy, enforce uniform types within columns or arrays. These systems do not permit mixed-type columns, making them incompatible with SQLite tables that include such heterogeneity.

To handle this mismatch, we implemented a workaround in CytoTable that dynamically constructs SQL queries using complex CASE expressions when exceptions related to this issue occur. These expressions detect and filter values based on SQLite's internal storage classes, allowing us to extract consistent, type-safe subsets of data. The NOT NULL constraint inhibited us from using potentially more efficient value replacement operations on the columns. While DuckDB performs most SQL operations in CytoTable are performed for its performance and analytical capabilities, these specific queries had to be executed within SQLite itself due to DuckDB's stricter typing enforcement. Although this solution was effective, it introduced significant engineering overhead and does not perform well due to the operating constraints. Based on this experience, we advise against using SQLite as a long-term data storage format for image-based profiling workflows, particularly because consistent data types are critical for performance, validation, and downstream tool compatibility.

Data type precision opportunities in image-based profiling

In image-based profiling workflows, numerical precision (particularly when storing floating point values) is sometimes an overlooked aspect of data integrity. Common formats such as CSV, SQLite, and NumPy offer limited control over numeric precision and type representation. For example, NumPy arrays are typically constrained to fixed-precision types like float64, and SQLite's type affinity model treats numeric fields flexibly, often mixing strings and floats in the same column. These formats may silently truncate or round values, which can pose issues for reproducibility and downstream analysis, particularly when subtle differences in feature magnitudes are biologically meaningful.

In contrast, modern data systems such as DuckDB and Apache Arrow offer more explicit and robust support for high-precision decimals. As shown in the accompanying notebook (please see "explore_floating_point_precision.ipynb" notebook within CytoTable-benchmarks repository [S6]), these systems allow for the definition and preservation of decimal types (e.g., decimal128(17, 16)), ensuring consistency across in-memory operations and persistent storage formats like Parquet. This level of precision could be important for HCI data where operations like normalization, aggregation, or phenotype scoring rely on subtle value differences. By preserving exact decimal representations, these platforms

help maintain data fidelity and enable more accurate downstream modeling, making them increasingly attractive for use in open-source profiling workflows.

As tools in the profiling ecosystem evolve, attention to numeric precision could become a larger concern to help create opportunities for profiling. Choosing formats and engines that enforce or respect explicit numeric types (e.g., Arrow-backed DataFrames and DuckDB SQL engines) can standardize the representation of values across workflows and improve confidence in analytical results. CytoTable's support for Arrow and DuckDB aligns with this principle, ensuring that profiling outputs retain their intended precision across the full data lifecycle, from in-memory processing to persisted datasets.

Apache Arrow memory management considerations

Apache Arrow, through its Python bindings in PyArrow, employs a high-performance, off-heap memory architecture that enables fast, zero-copy data access across language boundaries. Instead of using Python's traditional object memory management system (i.e., the Python heap), PyArrow often allocates large contiguous blocks of memory through a native memory pool. These allocations happen outside the purview of Python's garbage collector (GC), which allows for efficient sharing of columnar data between Arrow, NumPy, DuckDB, and other systems without serialization overhead. The allocator (whether malloc, jemalloc, or mimalloc) can be controlled through the ARROW_DEFAULT_MEMORY_POOL environment variable, which influences how memory is reserved and reclaimed during processing.

While this memory model offers substantial performance benefits for large-scale data workflows, we observed complications when using PyArrow within multithreaded or multiprocessed environments during CytoTable development. Specifically, PyArrow-allocated memory appeared to not always be released in a timely manner, especially under high-throughput conditions involving large batches of Arrow Tables. In several instances, the Python GC struggled to detect and free unused memory buffers, likely due to the lack of tight integration between Python's GC and Arrow's native memory pools. This led to unexpectedly high heap memory usage within memory profile reports, and in extreme cases, appeared to lead to memory exhaustion. Apache Arrow's memory management differs from what is found within CPython and as a result likely is the cause for these discrepancies.

To mitigate these issues, we experimented with different memory allocators and batch sizes, and in some cases introduced manual deallocation patterns or forced GC cycles. We found that the choice of memory pool (controlled via ARROW_DEFAULT_MEMORY_POOL environment variable) could significantly impact both observed performance and memory stability. While PyArrow's design is well-suited for high-performance analytical computing, users integrating it into complex pipelines, such as those used in image-based profiling, should remain aware of these edge cases and monitor memory usage closely when scaling up to threaded or distributed workloads.

References

S1. Shearer, C., and The, C.-D. (2000). The CRISP-DM Model: The New Blueprint for Data Mining. J. Data Warehous 5, 13–22.

S2. Martinez-Plumed, F., Contreras-Ochando, L., Ferri, C., Hernandez-Orallo, J., Kull, M., Lachiche, N., Ramirez-Quintana, M.J., and Flach, P. (2021). CRISP-DM twenty years later: From data mining processes to data science trajectories. IEEE Trans. Knowl. Data Eng. 33, 3048–3061.

S3. McKinney, W. (2010). Data Structures for Statistical Computing in Python. In Proceedings of the Python in Science Conference (SciPy), pp. 56–61.

S4. Datatypes In SQLite https://www.sqlite.org/datatype3.html.

S5. Constants — NumPy v2.4 manual https://numpy.org/doc/stable/reference/constants.html.

S6. Bunten, D., Lippincott, M., Alquaddoomi, F., and Way, G. (2025). CytoTable-benchmarks (Zenodo) https://doi.org/10.5281/ZENODO.15425830.
